# Pelvic lymph node dissection in high-risk prostate cancer

**DOI:** 10.1590/S1677-5538.IBJU.2020.1063

**Published:** 2021-02-28

**Authors:** Luciano Haiquel, Xavier Cathelineau, Rafael Sanchez-Salas, Petr Macek, Fernando Secin

**Affiliations:** 1 Sanatorio Las Lomas de San Isidro Department of Urology Buenos Aires Argentina Department of Urology, Sanatorio Las Lomas de San Isidro, Buenos Aires, Argentina; 2 Université Paris Descartes L’Institut Mutualiste Montsouris Department of Urology Paris France Department of Urology, L’Institut Mutualiste Montsouris, Université Paris Descartes, Paris, France; 3 Universidad de Buenos Aires Discipline of Urology Argentina Discipline of Urology, Universidad de Buenos Aires, Argentina

**Keywords:** Prostate cancer, familial [Supplementary Concept], Lymph Node Excision, Lymph

## Abstract

**Introduction:**

The therapeutic role of pelvic lymph node dissection (PLND) in prostate cancer (PCa) is unknown due to absence of randomized trials.

**Objective:**

to present a critical review on the therapeutic benefits of PLND in high risk localized PCa patients.

**Materials and Methods:**

A search of the literature on PLND was performed using PubMed, Cochrane, and Medline database. Articles obtained regarding diagnostic imaging and sentinel lymph node dissection, PLND extension, impact of PLND on survival, PLND in node positive “only” disease and PLND surgical risks were critically reviewed.

**Results:**

High-risk PCa commonly develops metastases. In these patients, the possibility of presenting lymph node disease is high. Thus, extended PLND during radical prostatectomy may be recommended in selected patients with localized high-risk PCa for both accurate staging and therapeutic intent. Although recent advances in detecting patients with lymph node involvement (LNI) with novel imaging and sentinel node dissection, extended PLND continues to be the most accurate method to stage lymph node disease, which may be related to the number of nodes removed. However, extended PLND increases surgical time, with potential impact on perioperative complications, hospital length of stay, rehospitalization and healthcare costs. Controversy persists on its therapeutic benefit, particularly in patients with high node burden.

**Conclusion:**

The impact of PLND on biochemical recurrence and PCa survival is unclear yet. Selection of patients may benefit from extended PLND but the challenge remains to identify them accurately. Only prospective randomized study would answer the precise role of PLND in high-risk pelvis confined PCa patients.

## INTRODUCTION

PCa is the second most frequent malignancy in men worldwide (1) and most patients have low-risk features (2, 3). However, 15% of men develop high-risk PCa and are more likely to metastasize and die from disease (4). Although patients with low-risk prostate cancer have a greater benefit with radical prostatectomy, a subset of patients with high-risk prostate cancer appears to benefit from surgical treatment (5).

The AUA/ASTRO/SUO guideline recommends radical prostatectomy as one of the standard treatments in this group and this should be accompanied by PLND assuming an estimated 15-40% LNI rate (6). PLND candidates may be selected based on clinical information such as serum PSA levels, Gleason score and estimated tumor volume (5, 7).

Memorial Sloan Kettering Cancer Center (MSKCC) and Briganti have developed nomograms to predict LNI in patients with localized PCa (6, 8). The EAU-ESTRO-SIOG and NCCN guidelines (1, 2) recommend the use of these predictive tools to select candidates for PLND. Of all the validated models, the Briganti and MSKCC nomograms were identified as the most accurate models available to predict LNI and have been validated by several authors showing comparable results (9, 10). However, some guidelines indicate that Partin tables and Roach formula could be useful as well (9).

Guidelines recommendations to select patient for PLND are not uniform. The European Urological Association (EAU) recommends PLND in patients with LNI risk >5% as per Briganti's nomogram (2, 11), while the AUA guidelines establish a 2% risk cut-off (12, 13).

Although extended PLND (ePLND) still represents the most accurate method for staging pelvis confined PCa (2), its therapeutic benefit is still unclear as ePLND increases surgical time and perioperative complications (14).

## MATERIAL AND METHODS

This critical review complied with some Amstar checklist criteria. We included PubMed, Cochrane, and Medline publications in the English language. Each subheading of this manuscript was framed and discussed according to ‘’PICO’’ (population, interventions, control groups and outcomes) principles. The authors verified in detail title, abstract, full text and data extraction of studies under review.

We included several retrospectives studies, reviews, comparative studies with at least one control arm and one meta-analysis. Comparative single-center cases and one ongoing clinical trial were included as well. Single case series, case reports, reviews, and editorial comments were excluded.

**Figure 1:**
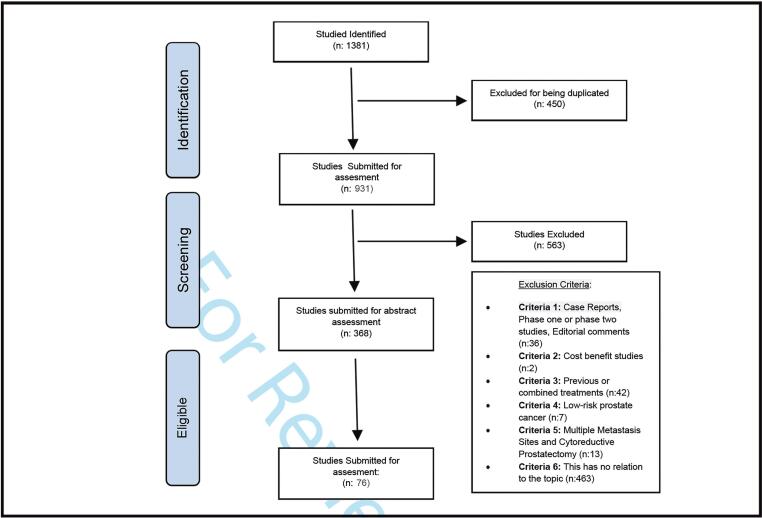
Flowcharts

Limitations of this study included lack of statistical analysis of crude data base information from different publications. In addition, this critical review includes retrospective studies as well. All authors deny conflicts of interest (Figure-1).

### Patient selection: diagnostic images and sentinel lymph node dissection

Unfortunately, the accuracy of routine images such as computed tomography or magnetic resonance imaging (MRI) to identify LNI is poor (15). Although increased node size is specific for LNI, its sensitivity is low (32%) as normal size nodes can harbor small foci of metastatic disease (16).

A synthetic ultrasmall superparamagnetic iron oxide composed of dextran-coated iron oxide nanoparticles known as Ferumoxtran-10 accumulates in non-cancerous lymphatic tissue, and has been used as a molecular MRI contrast agent. The administration of ferumoxtran-10 during MRI (17–20) has shown reliable differentiation of benign from malignant nodes, reaching a negative sensitivity and predictive value of 82% and 96% respectively (15). However, these studies have been limited by the low number of patients.

Although molecular imaging has emerged as a promising technique for improved lymph node staging in patients with PCa, positron emission tomography (PET) tracers like F18- or C-11-choline have not proved to be superior to anatomical imaging methods (20, 21) and a prospective study with C-11 choline PET/CT has shown disappointing results (22).

That said, some reports have shown encouraging data with PSMA PET/CT for initial detection of LNI. Recently, Ga-68 PSMA PET/CT has been proposed for the evaluation of biochemical recurrence after primary active treatment of PCa. Several studies have shown high diagnostic performance with a higher sensitivity compared to other radiopharmaceuticals such as radiolabeled choline (23, 24). However, a recent meta-analysis concluded that radiolabeled PSMA PET/CT shows a moderate sensitivity and high specificity for detection of metastasis and further studies would be necessary to substantiate the diagnostic accuracy of PSMA PET/TC for this purpose (25). Nonetheless, preoperative LN staging with 68Ga PSMA PET proved to be superior to standard routine imaging in patients with intermediate to high-risk PCa (26). In the absence of compelling evidence, its high costs and widespread inapplicability limit its routine use.

Increasing body of literature shows the utility of intraoperative PET gamma probes to detect LNI in head and neck, breast and endometrial cancers (27–30). Though promising, its benefit in prostate cancer surgery remains to be determined (31).

Since the initial works of Wawroshek et al. on sentinel lymph node dissection (SLND) in PCa (32) in 1999, there is still no consensus on its definition, technique, diagnostic role and utility. During a consensus group meeting on SLND, experts agreed that All nodes that appear first in each drainage basin seen early on (15 minute) lymphoscintigrams and/or single-photon emission CT imaging in new basins that were not yet seen on the early images^‥^ correspond to sentinel node (33). Experts also agreed on the potential utility of tracers with green indocyanine and a hybrid marker of technetium-99m nanocolloid (99mTc/green indocyanine) administered transrectally with a time interval ranging from 8 hours to 30 minutes before surgery according to the marker implemented (33). In a retrospective study, using data from 130 patients with intermediate to high-risk PCa, researchers found an estimated overall median sensitivity of 95.2%, which is promising and appears to be nearly twice as high as the average sensitivity reported for 11C- and 18F-choline and 68Ga-PSMA PET/CT (26).

Wit et al. reported that sentinel node biopsy (SNB) in PCa has almost equivalent diagnostic accuracy to ePLND, and recommends combining SNB with ePLND in high-risk disease (34). However, the false negative rate of SNB may range from 4.1% to 25% (35), and this highlights the importance of combining both procedures to improve staging.

Despite vast improvements in imaging studies, their combination with SNLD has been proposed to improve LND results. However, there is a need to define, optimize and standardize the technique for widespread applicability. PLND currently represents the gold standard for evaluating the presence of LNI.

### Lymphadenectomy, limited or extended?

**Figure 2:**
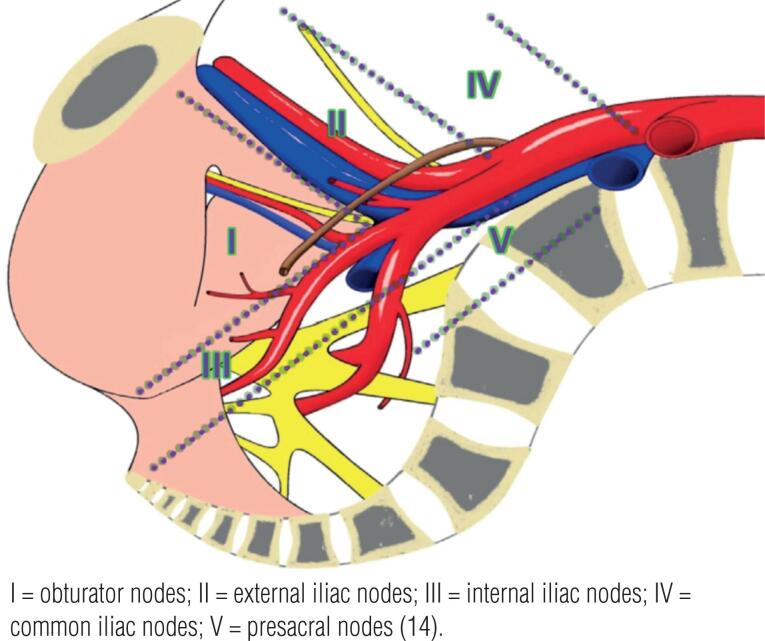
Anatomical areas for the definition of the extent of dissection.

While there is no unified definition, the EAU PCa Guideline Panel categorized PLND extension as (14) (Figure-2):

1. None;
2. Limited (lPLND): obturator nodes;
3. Standard (sPLND): obturator and external iliac nodes;
4. Extended (ePLND): obturator, external, and internal iliac nodes;
5. Super-extended (sePLND): ePLND plus common iliac, presacral, and/or other nodes;
6. Undefined or unclassified.

LNI rate seems to be related not only to disease biology but also to the extent of PLND. Several studies suggest there is a positive correlation between extent and number of pelvic nodes examined and metastatic burden (36–38).

In an attempt to establish the minimum number of nodes required during a prostatectomy, Barth et al. (39) recommended removing at least 13 nodes during sPLND. Interestingly, LNI rate doubled when more than 12 lymph nodes were examined. In addition, tissue work up method and handling by the pathologist appeared as an important factor for LNI diagnosis.

Briganti et al. (36) found that the possibility of detecting LNI was close to zero when less than 10 lymph nodes were removed in patients with a LNI risk ≥2% undergoing a PLND with a template that included the external iliac, obturator, and hypogastric lymph node packets. The authors provided a critical assessment of the concept that nodal yield at PLND is closely associated with LNI rate.

Abdollah et al. also estimated a count of 20 LNs for optimal pelvic lymph node staging in PCa as it renders a 10% false negative rate regardless of tumor characteristics (40). Heidenreich et al. recommended ePLND after they found that excluding internal iliac LND during PLND would leave behind 25% of all positive LNs (41). Their study also indicated close relationship between the number of LN removed and the long-term oncological outcome.

Weingärtner K et al. (42) performed sPLND on 30 human cadavers and 59 consecutive patients with clinically organ confined PCa during radical retropubic prostatectomy. PLND technique was performed in exactly the same manner in the human cadavers as in patients undergoing radical prostatectomy. The authors compared node count, size and distribution of all removed lymph nodes for each anatomical region in both groups. They concluded that approximately 20 pelvic lymph nodes would be an adequate sample size during a sPLND.

Mattei A. et al. informed that removal of LN around external iliac, hypogastric, obturator and common iliac LNs up to the ureteric crossing would remove approximately 75% of all nodes potentially harbouring metastasis (43).

Nodal metastases do not follow a predefined route, which is why, the more extended the PLND the higher the likelihood of metastases identification. Therefore, PLND should be as thorough as possible when clinically indicated.

### What is the impact of plnd on survival?

Literature is controversial and no adequate study has been developed to answer this question. Although several retrospective studies support the notion that ePLND may increase survival in high-risk PCa (44–48), recent results of a single institution double-arm prospective phase III study indicated the opposite. The authors found that ePLND had no impact on relapse and cancer specific survival (CSS). Table-1 summarizes the results of most recent publications.

**Table 1:** Baseline characteristics for studies addressing: Author and Study Date, type of study, numbers of patients and Features, surgical route, D'amico Risk or Gleason score, PSA and reports on oncologic outcomes.

Author, Study Date. Type of study	N° of Patients and Features	Treatment	Surgical route	D'amico Risk or Gleason score	PSA	Outcome reported on	Oncologic outcomes
Fossati, et al. (2017). Retrosperctive study. (Meta-analysis) Oncological outcomes by 29 studies (14).	-Experimental arm and one control arm; Studies with more than two arms T1-3 N0 M0 PCa	lPLND vs. ePLND vs. sePLND	ORP, RARP, LRP	**D'amico risk:** -Low -Intermediate -High	**NA**	**CSS, BCR**	**-**
Choo, at el. (2017). Retrospective study. (Meta-analysis). 2004-2014 (46).	Seven studies include to oncology outcomes results 1095 p.	sPLND vs. ePLND	RARP, ORP	**D'amico risk:** -Intermediate -High	**NA**	**BCR** (HR 0.71, 95% CI 0.56-0.90, p = 0.005)	**+**
Preisser (2017). Retrospective 2004-2014 (44). (SEER) database.	28147 patients.	lPLND (75%) vs. ePLND (24,8%)	NA	**Gleason** **- ≤6:** 2238 (8%) **- 7:** 19374 (68.8) **- ≥8:** 6535 (23.2%) **Intermediate risk** -18942 (67.3%) **High risk** -9205 (32.7%)	**Median PSA** **(IQR)** 6.5 (4.8-10)	**N°LN:** > 11 nodes removed improve 6-years PCa-specific survival (99.5% vs 98.1%, p: 0,014) **CSM-free:**ePLND: HR of 0.52 (C.I. 0.30-0.89, P = 0.017).	**+**
García-Perdomo et al. (2018). (Meta-analysis) (45). Retrospective study.	4 studies were included to study BRFS.	Pca N0M0 sPLND vs. ePLND	RAPR (1 study), ORP (3 studies)	**In Two studies:** -Low, intermediate and high risk. **In other two studies:** -Intermediate and high risk	**NA**	**BRFS** Favours ePLND HR = 0.62, 95% CI (0.36, 0.87)	**+**
Huele at al. (2018). Retrospective study (53). 2000-2016	228 p. (9 patients were excluded). Roach formula: 2/3 x prostate-specific antigen [PSA]+[Gleason score - 6]x10 (75)	Staging PLND before primary RT in a single tertiary care center	ORP (50), LPP (96), RARP(73)	**Risk group classification:** -Intermediate: 41 (18.8%) -High: 126 (57.8%) -Very high (locally advanced): 51 (23.4%)	**NA**	**BCR, CSS, OS**	**-**
Furubayashi et al. (2019). Retrospective single center study (47). 1998-2013.	348 patients T1-3 N0 M0 PCa.	sPLND (70.9%,247/348) vs ePLND (29.1%,101/348)	ORP (100%)	**-Gleason:** ≤7 sPLND 171 (69.2%), ePLND 70 (69.3%) **-Gleason:** >8 PLND 76 (30.8%), ePLND 31 (30.7%) **-N°Lymph N.** sPLND: 13 (0-31), ePLND: 19 (5-40)	**Median PSA**: - 8.171 ng/mL (range, 0.8 to 39.413 ng/mL **- PSA ≤10:** sPLND 170 (68.8%) vs. ePLND 50 (49.5) **- PSA >10:** sLPND: 77 (31.2%) vs ePLND: 51 (50.5%)	N° LN, PSA failure	**+**
Chen et al. (2019). Retrospective study (50). SEER database 2010-2015	20,668 patients.	No PLND vs. PLND-	NA	**D'Amico risk stratifcation, n (%)** **-Low** NPLND 366 (8.6%) PLND 369 (2.3%) **- Intermediate.** NPLND 2658 (62.3%) PLND 7463 (45.5%) **- High.** NPLND 1243p (29.1%) PLND 8569p (52.2%)	**PSa ≤20:** -NPLND: 4039 (94.7%) -PLND: 14462 (88.2%) **PSA >20:** -NPLDN 228 (5.3%) -PLND 1939 (11.8%)	**CSS** (5-year CSS rate: 99.4% vs. 99.7%, p=0.479)	**-**
Tomisaki et al. (2019) 2004 – 2011. No comparative Retrospective Single center study (52).	Consecutive 146 patients (RP without PLND); - MSKCC nomogram	No PLND	NA	**Gleason score** < 6: 61p, 3+4: 42p., 4+3: 15p. >8: 28 p. D'Amico classification Low: 39 p., **Intermediate:** 59 p., High: 48 p.	**Initial PSA:** - 7.6 ng/mL **Median (IQR):** - 7.6 (5.5-12.2)	**BCR** (Not inferior to others reports)	**-**
Sood et al. (2020). Retrospective study (48) 2004-2015 National Cancer Database (NCDB).	311.061 P -Risk was calculated using the Godoy-nomogram. Follow-up was 54.0 (31.3-79.9)	lPLND or No PLND (84,1%) vs. ePLND (15,9%); N°Lymph Node (m) lPLND 2 vs. ePLND 14.	NA	D'Amico Intermediate and high rick prostate cancer.	**Median PSA:** lPLND psa: 5ng/mL vs ePLND psa: 6 ng/mL	**CSS** 7% incremental benefit in 10-year CSS per every additional LN removed (P = 0.02).	**+**
Preisser et al. (2020). Retrospective study (51). Multi-institutional data base (4 centers). 2000- 2017.	9.742 p.	No PLND vs. PLND A median of 14 lymph nodes (IQR 8-21) were removed.	NA	D'Amico intermediate and high risk prostate cancer.	NA	**BRFR** 60.4% vs 65.6% (p=0.07) **SMFS** 95.2% vs 96.4% (p=0.2).	**-**
Lestigni et al. (2020). Prospective fase III study(49). 2012-2016	300 p. median follow-up (61,4 months) Pca (> cT2b or > PSA 10 ng/mL or Gleason score >7)	ePLND vs lPLND (1:1). RP **N°LN:** ePLND (mean) 17 n. vs lPLND (mean) 3 n.	RARP (100%)	D'Amico Intermediate and High risk prostate cancer.	**Median PSA, ng/mL (IQR):** ePLND 10.5 (6.5-17) vs lPLND 10.4 (6.9-13.9)	**BRFS.** (HR 0.91, 95% CI 0.63-1.32, p = 0.6) Subgroup (short time analysis) **BRFS** was better: biopsy **ISUP GG3–GG5** who underwent ePLND	**-**

**ASCO** = American Society of Clinical Oncology; **PLND =** lymphadenectomy; **ePLND** = extended PLND; **lPLND** = limited PLND; **sPLND** = standart PLND; **sePLND** = super selective PLND; **IQR** = interquartile range; **NA** = not available; **BCR** = biochemical recurrence; **BF** = biochemical failure; **CSS** = cancer-specific survival; **OS** = Overall Survival; **CSM-F** = Cancer specific metastasis free; **BRFS** = Biochemical recurrence – free survival.

In a systematic review, Fossati et al. evaluated 21 retrospective studies analysing the impact of PLND on oncologic outcome. The authors included 18 studies evaluating biochemical recurrence and six looking into survival in intermediate and high-risk PCa patients. This meta-analysis failed to demonstrate a therapeutic benefit of PLND (14).

In addition, several current retrospective studies have criticized the impact of PLND on survival. In one of them, 20.668 Surveillance, Epidemiology, and End results (SEER) database patients with PCa and >5% LNI risk as per Briganti's nomogram were evaluated. This study compared radical prostatectomy with and without PLND. No significant difference of CSS and overall survival (OS) was found between groups (5-year CSS rate: 99.4% vs. 99.7%, p=0.479, 5-year OS rate: 97.3% vs. 97.8%, p=0.204). They concluded that neither PLND nor its extension was associated with improved survival in these patients and perhaps the cut-off point of 5% is too low to show benefits in patients who underwent PLND along with the prostatectomy (50).

Preisser et al. compared oncologic outcomes of 9.742 intermediate and highrisk patients who underwent radical prostatectomy with or without PLND. The authors did not find significant differences in BCR (51).

Conversely, again Preisser et al. evaluated cancer specific mortality (CSM) in 28.147 SEER database PCa patients (2004-2014) subject to eLPND or lPLND. They found a lower CSM in patients who underwent eLPND. They estimated that removal of >11 nodes during PLND improved 6-year CSS and each additional LN removed reduced CSM risk by 4.5% (44).

In a retrospective meta-analysis involving 1.095 intermediate and high-risk PCa patients of 3 centers in Korea, Min Soo Choo et al. (46) suggested ePLND would provide oncological benefits by preventing biochemical relapse (BCR). The pooled analysis showed a significant reduction in BCR with ePLND compared to sPLND (HR 0.71, 95% CI 0.56-0.90, p=0.005).

An additional meta-analysis on 5 retrospectives studies (45), found improved BCR with ePLND, although two of them also included low-risk patients. Sood et al. recently evaluated 311.061 patients from the National Cancer Database (NCDB). They found that patients undergoing ePLND had 9% lower risk of 10-year mortality as compared to patients undergoing none or limited PLND (48).

Recent results of the only prospective phase III trial were presented. To define the primary endpoint, the authors compared biomedical recurrence-free survival (BRFS) among 300 patients with D'Amico's intermediate or high-risk PCa, who had received lPLND (n 150) or ePLND (n 150) during robotic prostatectomy. This trial was designed with 80% power and an alpha error of 0.05 to detect a 15% difference in 5-year BRFS. ePLND and lPLND yielded median (mean) 17 (19.8) and 3 (4.1) positive nodes, respectively (p <0.001), while ePLND resulted in five times more lymph node metastases detection (p <0.001). However, over a median follow-up of 61.4 month the authors found no difference in BRFS (HR 0.91, 95% CI 0.63 - 1.32, p=0.6), distant metastases or death between groups (48). Although the primary endpoint has not been reached, a short time subgroup analysis suggests a benefit in BRFS for patients who underwent ePLND diagnosed with preoperative biopsy International Society of Urological Pathology (ISUP) grade groups 3-5 (HR 0.33, 95% CI 0.14-0.74, interaction p=0.007) (49).

Another two prospective randomized studies (NCT01555086) are being carried out to establish the impact of PLND on survival. The first of these is a German clinical trial that aims to show whether the extent of PLND could influence PSA progression over 5 years. While we continue to expect results, new data have not been presented since 2017. Currently, a second clinical trial has begun recruiting patients in Switzerland (NCT03921996), but it only comparing ePLND vs. no PLND in patients who undergo radical prostatectomy.

The impact of PLND on survival is still controversial and results of others prospective studies are eagerly awaited.

### What are the outcomes of radical prostatectomy and plnd in node positive “only” disease?

The decision to perform treatment in advanced cancers is based on the principle of tumor volume reduction and local disease control. Patients with initial diagnosis of PCa and LNI represent 1.3-12% and they have a strong correlation with death (54). LNI in PCa represents a heterogeneous group of patients with different prognoses, depending on tumor grade and number of lymph nodes involved. A recent retrospective study done in the United States demonstrated an increased incidence of node-positive PCa and this may be in part explained by a more frequent use of ePLND (55).

In the past, when LNI was found during the frozen section, radical prostatectomy was terminated (56), and the patient treated with hormones. Historically, patients diagnosed with PCa who had clinical LNI were treated as if they had systemic diseases. These patients were prescribed androgen deprivation therapy (ADT), even if only one lymph node was involved. More recently, Moschini et al. (57) retrospectively compared survival between patients with and without suspicious nodes on usual preoperative imaging studies. The authors found no differences in CSS and OS and concluded that suspicious nodes on preoperative imaging was not an absolute contraindication to RP in adequately selected and well-informed surgical candidates.

Georgios Gakis et al. (58) reported in 2014 the need to better define LNI on the grounds that there is an improved survival in patients with lymph node only metastasis who underwent radical prostatectomy and ePLND.

Several retrospective studies describe the benefits of PLND in patients with LNI only disease. In a retrospective study involving 315 pN1 PCa patients, (59) the relationship between the number of lymph nodes removed and CSM was studied. The authors found a positive correlation between the number of lymph nodes removed and CSS (HR: 1.03, p: 0.05). Similar findings were reported by Elio Mazzone et al. (60) with added benefit on OS.

Nonetheless, the role of radical prostatectomy and PLND in patients with LNI remains unclear. Some authors argue that surgical treatment improves staging facilitating subsequent multimodal treatments (61) or postpone the onset of systemic therapy (62).

An institutional retrospective review evaluated predictors of clinical recurrence (CR) in patients undergoing radical prostatectomy with PLND. Approximately 80% had received neoadjuvant or adjuvant ADT. LNI and Gleason score ≥8 were associated with an increased risk of CR. On the contrary, prognostic group 1-3 patients with only one increased nodule on preoperative imaging studies exhibited favorable oncological results with surgical therapy. The authors emphasized the need for adequate preoperative staging (63).

Although several studies showed encouraging results, the impact of PLND on survival in pelvic LNI only patients remains to be elucidated. However, progress was made in understanding the importance of adequate patient selection to identify those who would potentially benefit most from PLND.

### What are the risks of extend PLND?

PLND during radical prostatectomy is usually well tolerated with a relatively low complication rate (64). Nonetheless, ePLND may be technically challenging and could lead to perioperative complications. Stone et al. reported a strikingly higher complication rate when they compared laparoscopic ePLND to laparoscopic lPLND (35.9% vs. 2%, p <0.001) (65).

Similarly, Briganti et al. reported a three-fold increase complication rate and longer hospital stay in patients undergoing ePLND compared to lPLND, and this was directly related to the number of LN removed (66).

Hospital readmissions were also higher in patients undergoing PLND during robot-assisted prostatectomy (RARP). Patients undergoing RARP and PLND had re-admission rates of 4.4% in comparison to 0.8% of those without PLND (67). However, Heidenreich et al. found that the frequency and severity of intra and postoperative complications did not differ significantly between limited and ePLND (9% vs. 8.7%, respectively) (68).

Asymptomatic lymphocele is the most frequent complication after PLND (69–71). Asymptomatic lymphocele detection rate on imaging studies varies from 27 to 61% (70, 72). The incidence of symptomatic lymphocele is around 8% (73).

On occasions, lymphoceles may lead to deep vein thrombosis due to vein compression. If lymphoceles get infected, sepsis may duly follow requiring more aggressive treatment (74).

Capitanio et al. found in a prospective study of 552 patients, that the higher the number of lymph nodes removed (>20 nodes) and the elder the patients (>65 years) the higher the likelihood of symptomatic lymphoceles after radical prostatectomy (74).

**Figure 3:**
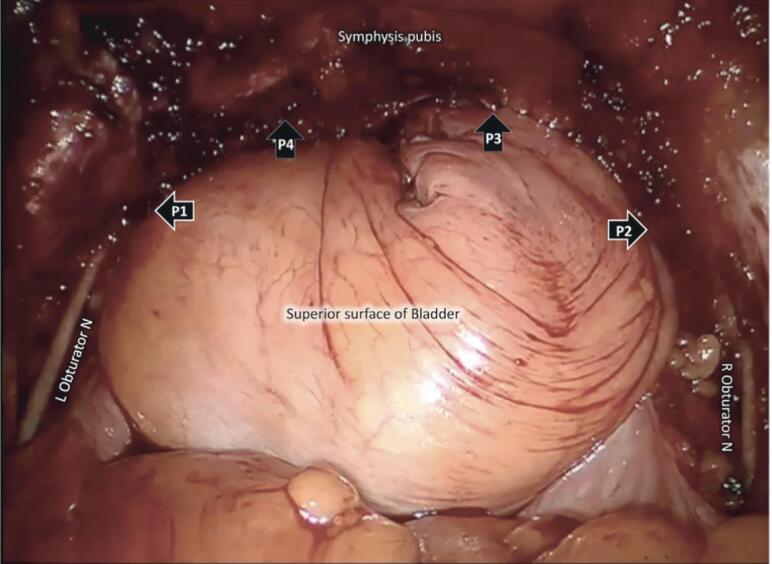
Status following completion of four-point peritoneal flap fixation (P1, P2, P3, P4). The obturator nerves on both sides can be visualized (75).

Improved surgical technique is key to diminish lymphocele rates. Stolzenburg, et al. proposed a technical modification consisting on suturing the cut end of the ventral parietal peritoneum back to the anterior and lateral pelvic side walls following ePLND to decrease the incidence of symptomatic lymphoceles, as shown in Figure-3 (75).

Another prospective study did not find significant differences in lymphocele rate when they compared the use of titanium clips to bipolar coagulation for the sealing of lymphatic vessels during RARP (76).

In sum, the more extended the PLND the higher the likelihood of perioperative complications, even in experienced hands. This should not refrain surgeons from performing ePLND when clinically indicated.

## CONCLUSIONS

Despite advances in imaging and sentinel node methods, ePLND remains the most accurate method for staging intermediate and high-risk PCa. The impact of ePLND on BCR and survival is unclear yet. Select patients may benefit from ePLND but the challenge remains to identify them accurately. Only prospective randomized studies would answer the precise role of PLND in intermediate and high-risk pelvis confined PCa patients.
